# The clinical acceptability of short versus long duration acquisitions for head and neck cancer using long-axial field-of-view PET/CT: a retrospective evaluation

**DOI:** 10.1007/s00259-023-06516-6

**Published:** 2023-12-14

**Authors:** Riccardo Mei, Thomas Pyka, Hasan Sari, Stefano Fanti, Ali Afshar-Oromieh, Roland Giger, Federico Caobelli, Axel Rominger, Ian Alberts

**Affiliations:** 1 Nuclear Medicine Department, IRCCS Azienda Ospedaliero-Universitaria Di Bologna, Bologna, Italy; 2 Department of Nuclear Medicine, Inselspital, Bern University Hospital, University of Bern, Freiburgstr. 18, 3010 Bern, Switzerland; 3 Advanced Clinical Imaging Technology, Siemens Healthcare AG, Lausanne, Switzerland; 4 Department of Head and Neck Surgery, Inselspital, Bern University Hospital, University of Bern, Bern, Switzerland; 5 Molecular Imaging and Therapy, BC Cancer Agency, Vancouver, BC Canada

**Keywords:** Total body, Ultra-long FOV PET, Whole body, PET/CT, Positron emission tomography, Digital PET, Head and neck cancers

## Abstract

**Purpose:**

To evaluate the utility of long duration (10 min) acquisitions compared to standard 4 min scans in the evaluation of head and neck cancer (HNC) using a long-axial field-of-view (LAFOV) system in 2-[18F]FDG PET/CT.

**Methods:**

HNC patients undergoing LAFOV PET/CT were included retrospectively according to a predefined sample size calculation. For each acquisition, FDG avid lymph nodes (LN) which were highly probable or equivocal for malignancy were identified by two board certified nuclear medicine physicians in consensus. The aim of this study was to establish the clinical acceptability of short-duration (4 min, C_40%_) acquisitions compared to full-count (10 min, C_100%_) in terms of the detection of LN metastases in HNC. Secondary endpoints were the positive predictive value for LN status (PPV) and comparison of SUV_max_ at C_40%_ and C_100%_. Histology reports or confirmatory imaging were the reference standard.

**Results:**

A total of 1218 records were screened and target recruitment was met with *n* = 64 HNC patients undergoing LAFOV. Median age was 65 years (IQR: 59–73). At C_40%_, a total of 387 lesions were detected (highly probable LN *n* = 274 and equivocal *n* = 113. The total number of lesions detected at C_100%_ acquisition was 439, of them 291 (66%) highly probable LN and 148 (34%) equivocal. Detection rate between the two acquisitions did not demonstrate any significant differences (Pearson’s Chi-Square test, *p* = 0.792). Sensitivity, specificity, PPV, NPV and accuracy for C_40%_ were 83%, 44%, 55%, 76% and 36%, whilst for C_100%_ were 85%, 56%, 55%, 85% and 43%, respectively. The improved accuracy reached borderline significance (*p* = 0.057). At the ROC analysis, lower SUVmax was identified for C_100%_ (3.5) compared to C_40%_ (4.5).

**Conclusion:**

In terms of LN detection, C_40%_ acquisitions showed no significant difference compared to the C_100%_ acquisitions. There was some improvement for lesions detection at C_100%_, with a small increment in accuracy reaching borderline significance, suggestive that the higher sensitivity afforded by LAFOV might translate to improved clinical performance in some patients.

## Introduction

The principle that glucose metabolism, as measured by the uptake of the positron emission radiotracer fluorodeoxyglucose (2-[^18^F]-FDG), could be used to measure the proliferative activity of head and neck cancers (HNC) was first established by Minn et al. in 1988 [1] who subsequently established that changes in uptake correlated with treatment outcome after radiotherapy [2]. Accordingly, combined positron emission and computed tomography (PET/CT) has become a well-established tool in the staging and evaluation of HNC, particularly in advanced stage disease [3]. Now in its third decade, PET/CT continues to undergo rapid and important technological development. For example, the introduction of time-of-flight and fully-digital systems have furnished improvements in scanner sensitivity and resolution, with faster time of flight resolution, increased scanner sensitivity and image quality [4]. These systems can improve diagnostic performance [5–7].

Most recently, long-axial field of view (LAFOV) systems have been introduced onto the market. They exhibit substantially improved whole-body coverage, sensitivity and quantification, and can afford low-activity or ultra-fast scan protocols [8–13]. The total recovered signal from a lesion is a function of both applied activity and acquisition time as well as dependent upon scanner characteristics [14]. However, the clinical utility of any substantially higher sensitivity on lesion detectability has not yet been fully evaluated. For example, it has been hypothesised that the higher scanner sensitivity might yield higher detection rates or afford detection of pathology at earlier stages, as has previously been demonstrated for digital scanners [5], e.g. in the detection of micrometastases in lymph nodes (LN) [15, 16]. Alternatively, the higher sensitivity can be exploited to reduce scan time. For example, Sachpekidis et al. (2022) demonstrate in patients with malignant melanoma that a 5-min low-dose acquisition using a LAFOV system can deliver comparable results to a standard scan [17]. The optimal acquisition time and activity for HNC in LAFOV is yet to be established.

A number of early studies using analogue PET systems demonstrated the additional clinical value of a high-resolution acquisition of the head and neck (H&N) region, with improved LN Detection [18–20] and this is common practice in many centres [21], and our institutional standard when using our digital SAFOV system (Siemens Biograph Vision) is for a 4-min per bed position equivalent acquisition over the head and neck in continuous bed motion.

The ability to scan the entire torso in a single bed position using LAFOV coupled with improved detection geometries allows for acquisitions which would be unfeasibly long when using a short axial FOV (SAFOV) system. To yield equivalent results for our clinical standard 10-min acquisition on the LAFOV, an impracticable 88 min using the SAFOV Siemens Biograph Vision in continuous bed motion (CBM) would be required for equivalent FOV coverage. This coupled with the higher sensitivity of a LAFOV system owing to improved geometry results in substantially higher count statistics with low noise images. In determining the optimal acquisition protocol for HNC in LAFOV, the balance between a clinical benefit in scanning longer or with more activity with higher image quality must be balanced against the requirement to scan faster or more gently, with lower activity protocols.

The aim of this study was therefore to assess the clinical acceptability of standard-duration (4 min, C_40%_) acquisitions compared to full-count (10 min, C_100%_) long duration acquisitions using a LAFOV system in terms of the detection of LN metastases in HNC. In doing so, we also assess the potential additional clinical value of the long-duration acquisitions which LAFOV is now able to provide and to evaluate any differences in diagnostic accuracy therein.

## Materials and methods

### Patient cohort and imaging procedures

The first Siemens Biograph Vision Quadra (Siemens Healthineers) LAFOV system was installed at our centre in October 2020. Cognisant of previous work demonstrating a learning curve when encountering digital PET/CT systems [5], we exclude patients examined during the first 3 months of operation of the new scanner. We therefore screened all patients who underwent PET/CT at our centre between 01 Jan 2021 and 31 Dec 2021 This retrospective analysis was approved by the cantonal ethics committee (KEK 2022–00486). Inclusion criteria were: patients examined on the Biograph Vision Quadra (LAFOV) scanner and who were referred for the staging or restaging of known or suspected squamous cell carcinoma of the head and neck. Exclusion criteria were: patients with known second non-HNC malignancy, patients undergoing therapeutic monitoring of HNC-malignancy (e.g. post-radiotherapy evaluation) and patients who did not provide informed consent for the retrospective analysis of their healthcare related data.

All patients arrived in a fasted state (> 6 h), more than 4 h since the last administration of insulin and with a blood glucose < 11.0 mmol/l confirmed by venous sampling prior to the administration of a standard administration of 3 MBq/Kg of 2-[^18^F]FDG with image acquisition at 60 min post-injection in accordance with EANM Guidelines [22]. List-mode PET emission data were acquired in a single bed position (106 cm axial FOV) from skull vertex to mid thighs using a maximum ring difference (MRD) of 85 for 10 min (C_100%_). Vendor recommended reconstruction (using a point-spread function and time of flight algorithm) was performed. Acquisition parameters were as previously published and as per clinical routine [8]. Where clinically indicated, additional contrast-enhanced CT of the head and neck region was performed after the PET/CT. A second set of PET images were reconstructed using 0–4 min, representing 40% of the total counts (C_40%_). A 4-min acquisition was chosen as representative of our routine digital SAFOV protocol (using the Siemens Biograph Vision 600), where a 4 min per bed position equivalent acquisition in CBM is obtained over the head and neck region.

All images were reviewed by two board certified nuclear medicine physicians (first and last authors) in consensus. LN with higher than background uptake of the radiopharmaceutical were noted as being FDG-avid. Based on clinical *Gestalt* evaluation of the scans, LN were rated by both readers as either highly probable for LN metastasis or, in clinically unclear cases, as equivocal for LN metastasis, and reflects the method of reading scans under clinical routine. The maximum standardised uptake value (SUV_max_) was measured using a 40% iso-contour volume of interest using appropriate software (Siemens Syngo.Via) and which has previously been described [23]. The total number of lymph nodes rated as highly probable for lymph node metastasis was recorded (nodal detection rate).

### Study design and sample size calculation

The primary endpoint was the total number of highly probable LN metastases identified at PET/CT for each reconstruction (i.e. the nodal detection rate). The secondary endpoints were the positive predictive value for lymph node status (PPV) and comparison of maximum standardised uptake value (SUV_max_) at C_40%_ and C_100%_. Additional outcomes of interest were the rate of indeterminate or equivocal findings, and test performance (sensitivity, specificity, NPV and accuracy). Null hypothesis (H_0_) was that there is no difference in terms of lesion detection between the two reconstructions, the alternative hypothesis (H_a_) is that more pathological LN are detected at C_100%_ compared to C_40%_.

The sample size was determined a priori and powered to test the hypothesis. A power calculation was performed to detect a treatment difference with a two-tailed alpha 0.05, Power 80%, minimum allowable difference in mean = 0.5 and conservative estimate of within-population variation = 3 [24]. A sample size of *N* = 64 was calculated [25].

### Follow up procedures

Follow up was performed with scrutiny of electronic clinical records of all patients for histopathology or correlative imaging data to confirm or refute findings. For patients who underwent neck dissection (*N* = 53), histopathology records were scrutinised and compared to the PET findings. Positive nodal status at histopathology was noted and the reported anatomical LN level (as per the American Joint Committee on Cancer AJCC anatomical lymph node levels, 8^th^ Edition) compared with the scan positive findings (definite and equivocal) to determine a per-region based true positive (TP) and false positive (FP) status.

### Statistical analysis

The frequency of lymph nodes rated as pathological for the C_40%_ and C_100%_ scans were compared by means of the Chi-squared test. *p* values < 0.05 were considered significant. Lesion SUV_max_ was compared by the Mann–Whitney *U*-test following confirmation of normality by the Kolmogorov–Smirnov test. A contingency table of findings was compiled to compare sensitivity, specificity, positive and negative predictive values, and test accuracy with differences between C_40%_ and C_100%_ calculated by means of Fisher’s exact test. ROC analysis was performed for the assessment of diagnostic performance between the two acquisitions and for SUV cut-off values which might serve as predictors of malignancy.

## Results

### Patient characteristics

**Fig. 1:**
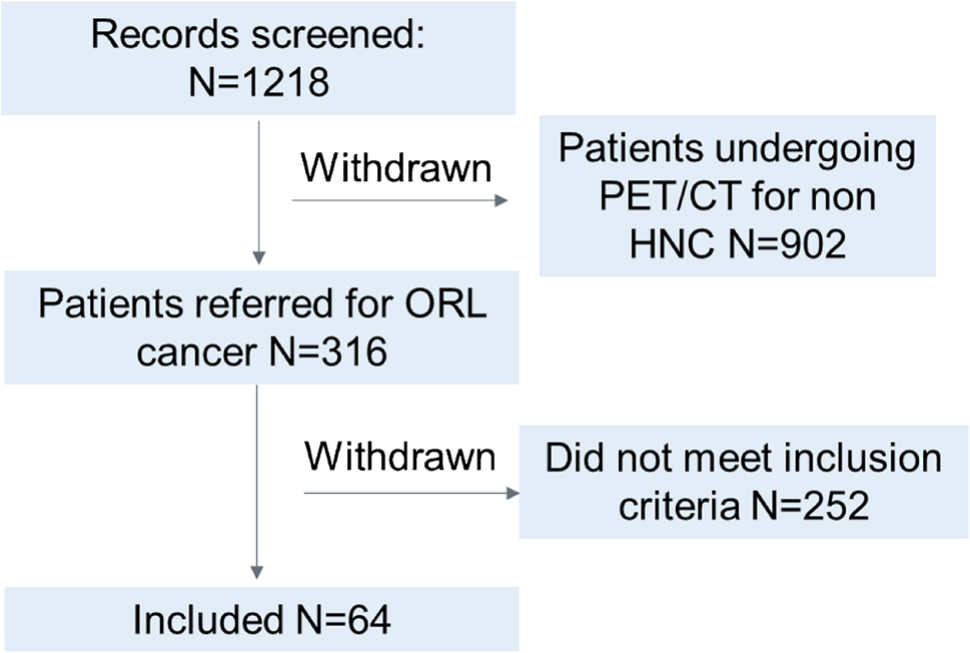
Study flow diagram. Screening failure is defined as those patients where one or more exclusion criteria applied, or who did not fulfil the inclusion criteria as described in the materials and methods section

All patients undergoing FDG PET/CT at our centre in 2021 were screened (1218 records). Target recruitment was met with *N* = 64 patients meeting the study inclusion criteria, of whom 42 were male and 22 female. Patient characteristics are as shown in Table 1. The study flow-chart is shown in Fig. 1. Indication for imaging was primary staging and restaging in 56 and 8 patients, respectively. Prior treatment before restaging was surgery in 6 patients and chemo-radiotherapy in 2. Median age was 65 years (IQR: 73–59). Median-injected activity was 221 ± 52 MBq. Histology reports were available in 53 patients.

**Table 1:** Patient characteristics. Data are *n* (%). Four-min (C_40%_) and 10-min (C_100%_) acquisition

	Overall (*n* = 64)
Age (years)	
Mean (SD)	66 (11)
Median [min, max]	65 [59-73]
Gender	
Female	22 (34.0%)
Male	42 (66.0%)
Indication	
*Initial staging*	56 (87.5%)
*Restaging*	8 (12.5%)
**Total lesions**	**C**_**40%**_** C**_**100%**_
Primary tumour	
*Equivocal* *Highly probable*	6 3 56 60
Nodal metastatic	
*Equivocal* *Highly probable*	96 140 191 185
Metastatic (organ and bone)	
*Equivocal* *Highly probable*	10 5 31 37

### Primary endpoint

At C_40%_ a total of 387 lesions were detected, of them 274 (70%) highly probable tumour lesions and 113 (30%) equivocal. The total number of lesions detected at C_100%_ acquisition was 439, of them 291 (66%) highly probable tumour lesions and 148 (34%) equivocal. The number of additional lesions at C_100%_ ranged 1–8 and was detected in 32 patients. Of these additional lesions, twelve were true positive findings, and false negatives were six. In 13 patients, C_40%_ detected additional lesions (range number 1–5). Detection rate between the two acquisitions did not demonstrate any difference (Pearson’s Chi-Square test, *p* = 0.792). As was expected, there was no statistically significant difference in SUVmax C_40%_ and C_100%_ (Mann–Whitney, *p* = 0.715). The results are shown in Table 2.

**Table 2:** Contingency table showing frequency of true positive, true negative, false positive and false negative findings at the per-region level for lymph nodes (LN)

LN histology	PET/CT findings			
	C_40%_		C_100%_	
	Negative	Positive	Negative	Positive
No LN metastases	54	17	96	17
LN metastases	68	84	76	93

### Secondary endpoints

In cases where patients underwent LN-dissection, nodal histopathology served as the gold-standard reference. The regional-level sensitivity, specificity, PPV, NPV and accuracy of the C_40_ acquisitions were 83%, 44%, 55%, 76% and 36% respectively. Conversely sensitivity, specificity, PPV, NPV and accuracy at C_100%_ were 85%, 56%, 55%, 85% and 43%, respectively, and are shown in Table 3. Fisher’s exact test revealed no significant differences in test accuracy parameters (sensitivity, PPV, NPV and accuracy) but higher specificity for C_100%_ with borderline significance (*p* = 0.0584).

**Table 3:** Diagnostic test parameters for 4-min (C_40%_) and 10-min (C_100%_) acquisitions compared by Fisher’s exact test

Lymph node test accuracy	C_40%_	C_100%_	*p* value
Sensitivity	83	85	0.517
Specificity	44	56	0.744
PPV	55	55	1.00
NPV	76	85	0.267
Accuracy	36	43	0.057

**Fig. 2:**
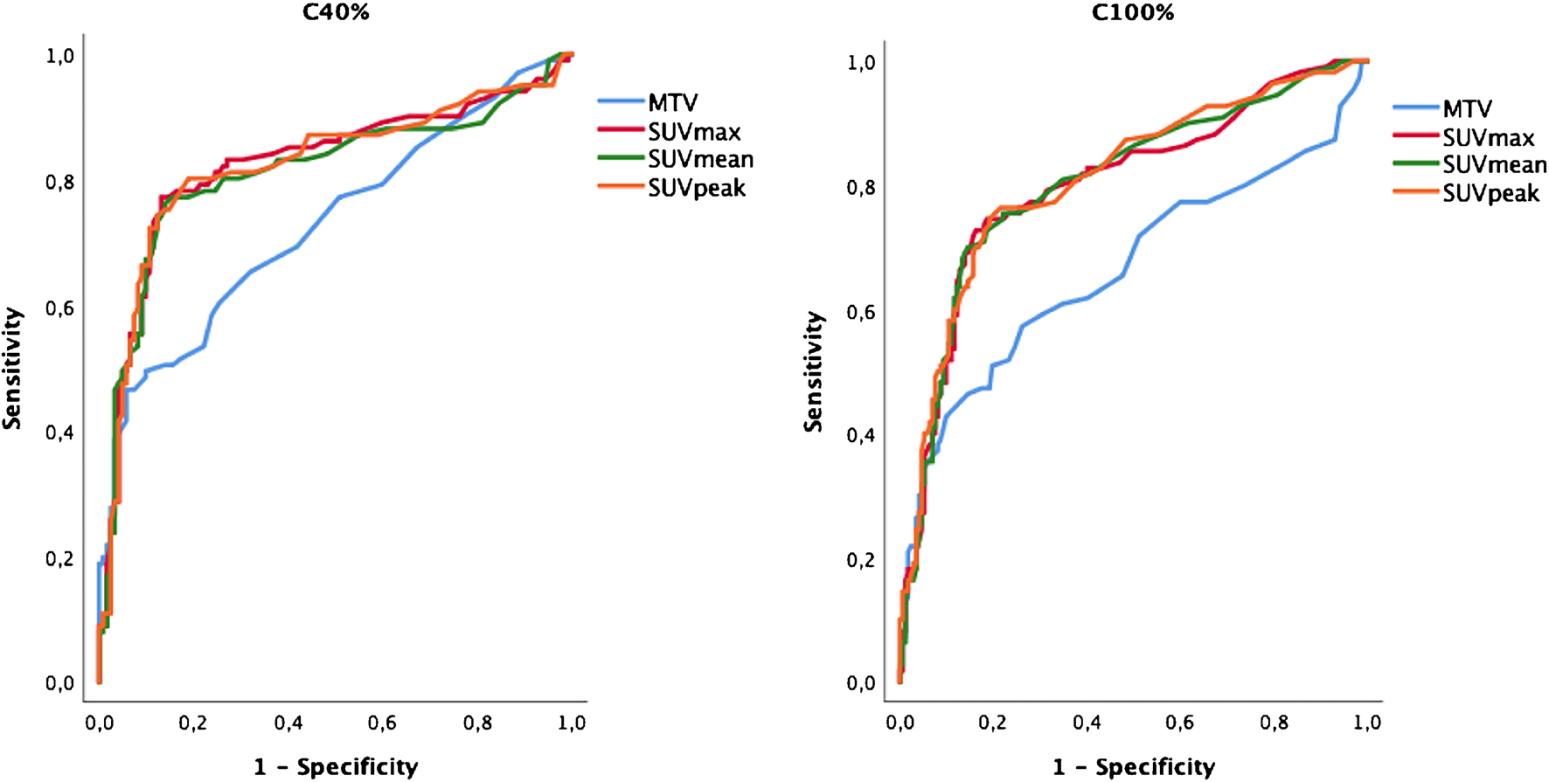
ROC curves showing performance of SUVs according to 4-min and 10-min acquisitions

At the ROC analysis, uptake values (SUVmax, SUVmean and SUVmean) showed similar AUCs both at C_40%_ and C_100%_ (mean AUC 0.75, see Fig. 2). We identified for C_40%_ an optimal SUVmax cut-off of 4.5 for with acceptable accuracy for detecting positive tumour lesions (sensitivity and specificity of 80% and 76%, respectively). Conversely, at C_100%_ the same accuracy was reached with a SUVmax cut-off value of 3.5.

As shown in Table 1, no significant different in rates of findings rated as equivocal were reported for primary lesions at C_40%_ versus C_100%_ (6/62 vs. 3/69, *p* = 0.3234). Statistically significant higher rates for reported for nodal lesions at C_100%_ (96/287 vs. 140/325, *p* = 0.0159).

**Fig. 3:**
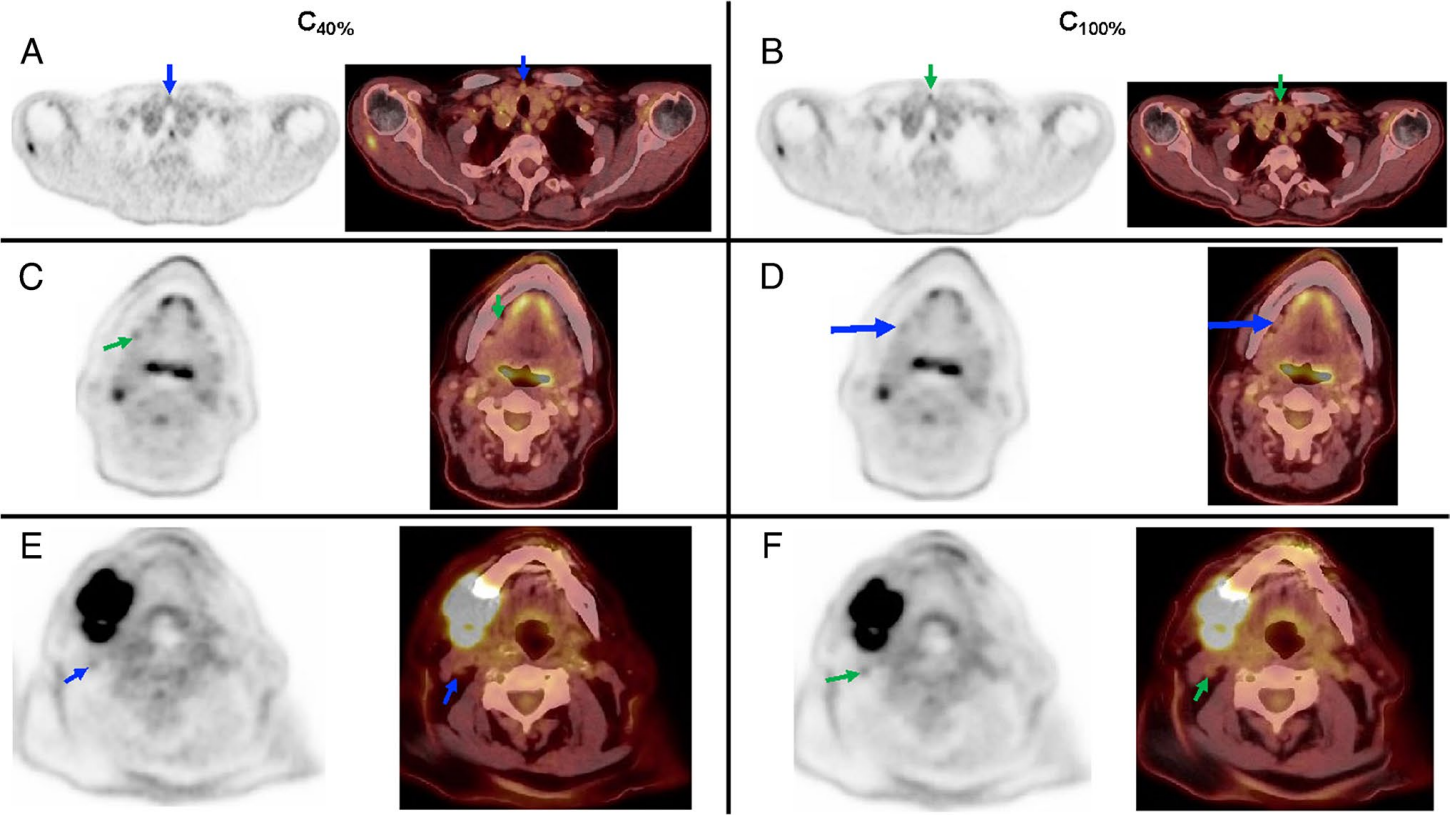
Example cases and positive lymph nodes (arrows) on the 4-min (C_40%_) and 10-min (C_100%_) acquisitions. **A**, **B** Patient with metastatic squamous cell oral carcinoma with a LN metastasis (level VI on the right), with better visualisation at C_100%_ acquisition (green arrow) rather than C_40%_ (blue arrow). **C**, **D** Patient with pharynx squamous cell carcinoma (SCC) shows multiple cervical LN metastases with a small submandibular lymph node with low FDG-uptake only at C_40%_ (green arrow), which was not appreciated in the C_100%_ (blue arrow). **E**, **F** A small lymph node adjacent to an enlarged node metastasis in the II level in a patient with oral cavity squamous cell carcinoma was detected only at C_100%_ (green arrow), with suboptimal visualisation at C_40%_ (blue arrow)

Example patient images are shown in Fig. 3. The cases show two examples where lymph node metastases were better visualised at C_100%_ compared to C_40%_ and one case where focal uptake can be appreciated at C_40%_, but is not seen at C_100%._

## Discussion

In this study, we find that a 4-min (C_40%_) acquisition and a 10-min (C_100%_) acquisition using standard activities on a LAFOV system show no significant differences in terms of lymph node detection in patients undergoing PET/CT for HNC. This demonstrates that, compared to standard activities and scan duration, substantial reductions in both are feasible and clinically acceptable with LAFOV systems. Whereas half-dose acquisitions with LAFOV have recently been shown to be acceptable in lung cancer [26], and low-dose 5-min acquisitions are suitable for melanoma [17], the optimal scan duration or applied activity for HNC with LAFOV is yet to be determined. This present study represents the first systematic evaluation of LAFOV PET/CT in HNC and is the first to assess the clinical value of the low noise high integral count activity examinations.

Alternatively, a 10-min scan could be performed and only 40% of the activity be applied, resulting in a reduction in radiation exposure from 4.3 to 1.7 mSv for a standard 75 kg adult patient [22], and further activity reductions might be possible without detriment to image quality [17]. Faster scans also might result in reduced patient movement, be more acceptable to patients with claustrophobia and allow for greater scanner throughput, widening access and shortening waiting times [27].

Although LAFOV systems allow for faster or gentler imaging with lower activities owing to their substantial higher sensitivity profiles through improved geometry [11, 28], the degree to which this additional scanner sensitivity allows for better or more accurate imaging is yet to be established. As was discussed earlier, longer-duration dedicated H&N-acquisitions on analogue systems showed improved detection of LN metastases in HNC [18–20]. Congruent to this finding, it might be postulated that the higher sensitivity of LAFOV systems might result in yet further improvements in diagnostic performance. For example, LAFOV might allow micro-metastases to be detected [15, 16]. Earlier and more accurate detection of pathology has already been demonstrated for digital silicon photomultiplier systems when compared to analogue systems [5–7, 29] but these findings are yet to be replicated with LAFOV systems. Although not powered as a superiority study, we did indeed find small but non-significant improvement in LN detection in the full count (C_100%_) scans, with additional lesions identified in 32 patients. The clinical vignettes shown in Fig. 2 show how the higher sensitivity afforded by full-count scans allow better detection of small LN metastases in HNC. Likewise, the lower noise at C_100%_ afforded better interpretation of a LN with low but focal avidity at the C_40%_. Follow-up imaging post radiotherapy, where the LN was outside of the radiation port and remained unchanged confirmed that this finding was a reactive lymph node. Likewise, small but borderline-significant improvements were observed in C_100%_ acquisitions for accuracy and ROC-analysis revealed a lower SUV cut-off. Taken together, we interpret these findings as suggestive that longer, full-count acquisitions provided some additional diagnostic benefit which translate to improved clinical performance. In-keeping with previous studies in digital PET, we find that this higher detection does translate to lower reader confidence with higher rates of equivocal findings at C_100%_ [5], although it is important to note that this was not at the expense of accuracy. When devising fast or low-activity protocols with resultant lower lesion integral activities, care should be taken that the potential benefits of high-quality full-count and low noise acquisitions are not forfeited.

The dual-time point evaluation of uptake kinetics has been investigated and might help differentiate between LN metastases and inflammatory LN in a number of entities [30–34]. Twenty-minute duration acquisitions have already been demonstrated to allow reliable analysis of tracer kinetics [35, 36]. We therefore wondered whether the high temporal resolution which LAFOV provides could provide insight into tracer kinetics over 10 min [35]. However, our data show no appreciable changes in SUV_max_. Conversely, reliable quantification was possible even in the space of a 4 min scan, where SUV_max_ can vary with noise and exposure [14].

This study has some limitations. The present study compares acquisitions after 4 min (chosen as the equivalent to a clinical standard SAFOV scan) and the full 10-min acquisition. Future studies might consider other acquisition durations and applied activities; a prospective study evaluating the non-inferiority of low-dose acquisitions is ongoing (NCT05496920). Future prospective studies might test any potential clinical advantage, including at the patient-outcome level to determine the clinical utility of LAFOV systems in HNC. Although our study was adequately powered to test a set of pre-defined statistical hypotheses, we recognise that 64 patients cannot test all circumstances where high-count acquisitions might be of additional utility, such as in the detection of systemic metastases, for which larger cohorts will be needed.

## Conclusion

This study demonstrated the clinical acceptability of 4 min acquisitions compared to 10-min acquisitions for the detection of LN metastases in HNC. Ten-minute acquisitions were associated with small and borderline-significant improvements in lesion detection and accuracy. We posit that the longer duration acquisitions which LAFOV systems afford might translate into improved diagnostic performance, which future studies might investigate.

## Data Availability

Data is available upon reasonable request.
